# Supplementary material for: Mechanisms governing the pioneering and redistribution capabilities of the non-classical pioneer PU.1
Source: Nat Commun. 2020 Jan 21;11:402. doi: 10.1038/s41467-019-13960-2 (PMC6972792; doi:10.1038/s41467-019-13960-2)
Supplement: Supplementary file 7 — Source data [file 41467_2019_13960_MOESM7_ESM.zip › Source_Data/Figure6/Figure6B_MotifScanOutput/FLI1_PU.1/homerResults/motif5.similar.html]

motif5

## Information for motif5

G
C
A
T
A
C
T
G
T
G
C
A
A
T
C
G
C
T
A
G
G
C
T
A
G
C
T
A
C
G
T
A
A
G
T
C
T
C
A
G
A
T
C
G
  
Reverse Opposite:  

T
A
G
C
A
G
T
C
T
C
A
G
A
C
G
T
C
G
A
T
C
G
A
T
G
A
T
C
T
A
G
C
A
C
G
T
T
G
A
C
C
G
A
T
  

|  |  |
| --- | --- |
| p-value: | 1e-330 |
| log p-value: | -7.621e+02 |
| Information Content per bp: | 1.522 |
| Number of Target Sequences with motif | 1780.0 |
| Percentage of Target Sequences with motif | 17.89% |
| Number of Background Sequences with motif | 1821.7 |
| Percentage of Background Sequences with motif | 4.99% |
| Average Position of motif in Targets | 103.7 +/- 53.8bp |
| Average Position of motif in Background | 103.0 +/- 66.7bp |
| Strand Bias (log2 ratio + to - strand density) | -0.1 |
| Multiplicity (# of sites on avg that occur together) | 1.00 |
| Motif File: | file (matrix) reverse opposite |

### Similar de novo motifs found

|  |  |  |  |  |  |  |  |
| --- | --- | --- | --- | --- | --- | --- | --- |
| Rank | Match Score | Redundant Motif | P-value | log P-value | % of Targets | % of Background | Motif file |
| 1 | 0.776 | G A T C C T G A C A T G T C A G T G C A G C T A T G C A A T G C G C A T T C G A | 1e-279 | -644.040981 | 17.69% | 5.62% | motif file (matrix) |
| 2 | 0.790 | A T G C A C T G G T A C C G T A A C T G A C T G G T C A C G T A G T C A A G T C | 1e-97 | -224.476264 | 4.78% | 1.15% | motif file (matrix) |
